# Editorial: Anaerobic Microorganisms on Mars 2.0

**DOI:** 10.3390/microorganisms11030734

**Published:** 2023-03-13

**Authors:** Felipe Gómez

**Affiliations:** Centro de Astrobiología (INTA-CSIC), Torrejón de Ardoz, 28850 Madrid, Spain; gomezgf@cab.inta-csic.es

Recent space missions (MSL-Curiosity, Mars2020-Perseverance) have confirmed the historic presence of water on early Mars [1]. Likewise, optimal habitability conditions have been reported to have existed on the red planet in the past [2]. Therefore, there was a real possibility of the development of an ecosystem on the subsurface of the planet. Could there be any record of that potential ecosystem, such as some kind of organic footprint or biosignature that we could find today?

The extreme environments characterized by these conditions, which are mainly anaerobic, are examples of terrestrial analogues that can provide us with clues to elucidate the possible biosignatures that we might search for, as well as the preservation potential of these biosignatures in such harsh conditions. Faced with this possibility, anaerobic organisms take the center stage due to the electron acceptors that they use in their respiratory chains. On the other hand, the high radiation incidence, as well as other factors that establish oxidizing conditions, could promote the low stability of organic molecules.

The relevant conditions that characterize these environments, which the supposed ecosystem would have also had to manage, are temperature (freezing temperatures), high radiation (with ultraviolet radiation playing an important role in the preservation of organic matter), the presence of chaotropic salts which promote very low levels of water activity, with important consequences for life, and low humidity similar to that observed in very dry deserts. The exploration of extreme environments with these above-described characteristics has led to the identification of some natural sites that were considered uninhabitable [3,4] until further research finally led to reports of the presence of life forms [5]. Subsequently, the study of these highly poli-extreme natural sites has increased research interests in the limits of life [4,6].

This special Issue of *Microorganisms* dedicated to the topic of “Anaerobic Microorganisms on Mars 2.0”, covers the different aspects described above and faced by life existing in these extreme environments. Starting with the complete absence of oxygen, the presence of chaotropic salts, cold temperatures, and/or high ultraviolet radiation are considered as critical parameters that can provide us with crucial clues so as to better understand the possible past environments on early Mars.

## References

[1] Grant J.A., Wilson S.A., Mangold N., Calef F., Grotzinger J.P. (2014). The timing of alluvial activity in Gale crater, Mars. Geophys. Res. Lett..

[2] Grotzinger J.P., Sumner D.Y., Kah L.C., Stack K., Gupta S., Edgar L., Rubin D., Lewis K., Schieber J., Mangold N. (2014). A habitable fluvio-lacustrine environment at Yellowknife Bay, Gale Crater, Mars. Science.

[3] Kotopoulou E., Delgado Huertas A., Garcia-Ruiz J.M., Dominguez-Vera J.M., Lopez-Garcia J.M., Guerra-Tschuschke I., Rull F. (2018). A Polyextreme hydrothermal system controlled by iron: The case of Dallol at the Afar triangle. ACS Earth Space Chem..

[4] Benison K.C., O´Neill W.K.O., Blain D., Hallsworth J.E. (2021). Water activities of acid brine lakes approach the limits for life. Astrobiology.

[5] Gómez F., Cavalazzi B., Rodríguez N., Amils R., Ori G.G., Olsson-Francis K., Escudero C., Martínez J.M., Miruts H. (2019). Ultra-small microorganisms in the polyextreme conditions of the Dallol volcano, Northern Afar, Ethiopia. Sci. Rep..

[6] Merino N., Aronson H.S., Bojanova D.P., Feyhl-Buska J., Wong M.L., Zhang S., Giovannelli D. (2019). Living at the Extremes: Extremophiles and the Limits of Life in a Planetary Context. Front. Microbiol..

